# Water Addition Prolonged the Length of the Growing Season of the Desert Shrub *Nitraria tangutorum* in a Temperate Desert

**DOI:** 10.3389/fpls.2020.01099

**Published:** 2020-07-21

**Authors:** Fang Bao, Minghu Liu, Yanli Cao, Jiazhu Li, Bin Yao, Zhiming Xin, Qi Lu, Bo Wu

**Affiliations:** ^1^ Institute of Desertification Studies, Chinese Academy of Forestry, Beijing, China; ^2^ Key Laboratory for Desert Ecosystem and Global Change, Chinese Academy of Forestry, Beijing, China; ^3^ Experimental Center of Desert Forestry, Chinese Academy of Forestry, Dengkou, China; ^4^ Inner Mongolia Dengkou Desert Ecosystem National Observation Research Station, Dengkou, China

**Keywords:** desert species, growing season, foliar phenology, branch phenology, water addition

## Abstract

Climate models often predict that more extreme precipitation events will occur in arid and semiarid regions, where plant phenology is particularly sensitive to precipitation changes. To understand how increases in precipitation affect plant phenology, this study conducted a manipulative field experiment in a desert ecosystem of northwest China. In this study, a long-term *in situ* water addition experiment was conducted in a temperate desert in northwestern China. The following five treatments were used: natural rain plus an additional 0, 25, 50, 75, and 100% of the local mean annual precipitation. A series of phenological events, including leaf unfolding (onset, 30%, 50%, and end of leaf unfolding), cessation of new branch elongation (30, 50, and 90%), and leaf coloration (80% of leaves turned yellow), of the locally dominant shrub *Nitraria tangutorum* were observed from 2012 to 2018. The results showed that on average, over the seven-year-study and in all treatments water addition treatments advanced the spring phenology (30% of leaf unfolding) by 1.29–3.00 days, but delayed the autumn phenology (80% of leaves turned yellow) by 1.18–11.82 days. Therefore, the length of the growing season was prolonged by 2.11–13.68 days, and autumn phenology contributed more than spring phenology. In addition, water addition treatments delayed the cessation of new branch elongation (90%) by 5.82–12.61 days, and nonlinear relationships were found between the leaves yellowing (80% of leaves) and the amount of watering. Linear relationships were found between the cessation of new branch elongation (90%), the length of the growing season, and amount of water addition. The two response patterns to water increase indicated that predictions of phenological events in the future should not be based on one trend only.

## Introduction

Plant phenology, i.e., the timing of seasonal life cycle events, such as leafing out and flowering, plays a fundamental role in the function of terrestrial ecosystems (Fu et al., 2014a; Fu et al., 2015; Browning et al., 2019). The timing of phenological events is strongly controlled by the prevailing climate and has long been regarded as one of the most sensitive and accurate bio-indicators to track climate change (Badeck et al., 2004; Bertin, 2008; Gordo and Sanz, 2010). Advancing the understanding of the phenological response to climate change is therefore important for forecasts of the impact of future climate change on terrestrial ecosystems (Cleland et al., 2007; Nord and Lynch, 2009). With the climate change observed over recent years, advances of spring phenology and delays of autumn phenology have been reported worldwide such as in Europe (Menzel and Fabian, 1999; Menzel et al., 2001; Fu et al., 2014a), North America (Schwartz and Reiter, 2000; Jeong et al., 2011; Fridley, 2012), the Southern Hemisphere (Chambers et al., 2013; Ma et al., 2013), and China (Ge et al., 2014; Ge et al., 2015; Zheng et al., 2016). This variation was attributed to prevailing climate warming trends (Cleland et al., 2007; Bertin, 2008). This is true for ecosystems without chronic seasonal water stress; however, the effects of temperature on vegetation phenology may be critically modulated in arid and semiarid ecosystems by soil water availability (Moore et al., 2015). Many studies have investigated how precipitation regulates plant phenology in seasonally dry tropical forests (Hayden et al., 2010) and other water-limited ecosystems (Patrick et al., 2009; Lesica and Kittelson, 2010; Liu et al., 2015; Zhou and Jia, 2016). For example, in a water-limited ecosystem in California, the rainfall volume and timing during winter jointly influenced the timing of vegetative bud break, where high rainfall in December and March delayed bud break, while high rainfall in February advanced bud break (Mazer et al., 2015). The degree of budburst and leaf extension was shows to be a function of irrigation intensity at the Estacion Biologia de Chamela in western Mexico (Hayden et al., 2010). Based on remote sensing data, preseason precipitation (i.e., precipitation before the growing season) was found to exert a stronger influence on the starting date of the vegetation growing season (SOS) of grasslands in drier areas than in wetter areas of the Qinghai–Tibetan Plateau (Shen et al., 2011; Shen et al., 2015). Moreover, the effect of total preseason precipitation on the end date of the growing season (EOS) in dry grasslands is greater than that of temperature for Inner Mongolia, China (Liu et al., 2015; Zhou and Jia, 2016). Similar results were also reported for arid and semiarid regions of Africa (Zhang et al., 2005; Gaughan et al., 2012). Shoot elongation during the growing season is an integral component of the annual sequence of developmental events in plants (Codesido and López, 2003). Shoot elongation rates were found to be related to rainfall in the two evergreen, woody, Brazilian Cerrado species *Leandra lacunose* and *Miconia albicans* (Damascos et al., 2005).

The phenological responses of plants in desert ecosystems in particular are causing increasing concern (Ghazahfar, 1997; Ogle and Reynolds, 2004; Leeuwen et al., 2010; Kigel et al., 2013; Sakkir et al., 2014; Yan et al., 2016; Huang et al., 2018). Desert ecosystems cover approximately 30% of the land surface and are strongly controlled by water availability (Patrick et al., 2009). Precipitation and water availability likely affect desert plant phenology stronger and more directly than the phenology of ecosystems with greater precipitation (Ghazahfar, 1997; Yan et al., 2016). Several studies have explored the responses of desert plant phenology to precipitation variation in their natural conditions. For example, the onset and duration of growth in chamaephytes and therophytes are highly correlated with both the timing and abundance of precipitation, whereas phanerophytes are least affected in the gravel desert of northern Oman (Ghazahfar, 1997) and in eastern United Arab Emirates (Sakkir et al., 2014). At the regional scale, spatial shifts in the onset of the vegetation growing season are controlled by summer rainfall in the southern Sahara Desert (Yan et al., 2016). Winter precipitation explained 14.2% of the inter-annual variations of spring phenology in the desert ecosystem of northwestern China (Wu and Liu, 2013).

Deserts across northwestern China cover an area of approximately 1.3 million km^2^ and are constantly expanding into neighboring ecosystems because of climatic changes and human activities (Huang et al., 2015a; Huang et al., 2015b). Future climate scenarios predict that the precipitation regimes in the desert regions of northwestern China will likely change, following an increasing trend (Gao et al., 2012; Chen, 2013; IPCC, 2014; Li et al., 2016; Wang et al., 2017). For example, based on the RCP8.5 scenario, increases in annual precipitation of 25%, 50% (Gao et al., 2012), and greater than 100% (Wang et al., 2017) of mean annual precipitation are expected in specific desert regions at the middle and end of the 21st Century compared with the end of the 20th Century (Song et al., 2020). Most of the biological processes in desert ecosystems are controlled by soil water availability, which is generally controled by rainfall events (Song et al., 2020). Precipitation increases likely impose substantial impacts on plant phenology in these deserts in response to climate change. Few studies examined the effects of water addition on reproductive phenology of six annuals on the southern fringe of the Gurbantunggut Desert in northwestern China and the results were only based on a short-time manipulative experiment (Huang et al., 2018). The results showed that water addition consistently advanced both the flowering and fruiting time of four spring ephemerals; however, their effects on two spring–summer annuals were inconsistent, where advances were found in one species, while delays were found in another (Huang et al., 2018). Since this study only focused on ephemerals, it remains unknown how the desert plant phenology of dominant perennials responds to an increase in precipitation, especially long-term precipitation increases.

To address this question, this study conducted a long-term *in situ* water addition experiment in a temperate desert of northwestern China, which is dominated by the shrub species *Nitraria tangutorum*. Five simulated future precipitation regimes (natural rain and natural rain plus an additional 25, 50, 75, and 100% of local long-term mean annual precipitation (145 mm)) were studied during the growing seasons from 2008 to 2018. A series of phenological events, including leaf unfolding (onset, 30%, 50%, and end of leaf unfolding), cessation of new branch elongation (30, 50, 90%), and leaf coloration (80% of leaves turned yellow) of *N. tangutorum* were assessed from 2012–2018. This *in situ* long-term water addition experiment provides accurate phenological information at the species level. The specific aims of this study were to: (i) clarify how increased water availability will affect phenological events (specifically, whether these will be advanced or delayed); (ii) identify how water increases affect the length of the growing season (specifically, whether it will be prolonged or shortened); (iii) determine how phenological events and the duration of the growing season respond to water addition (specifically, whether these responses will be linear or nonlinear).

## Materials and Methods

### Site Description

**Figure 1:**
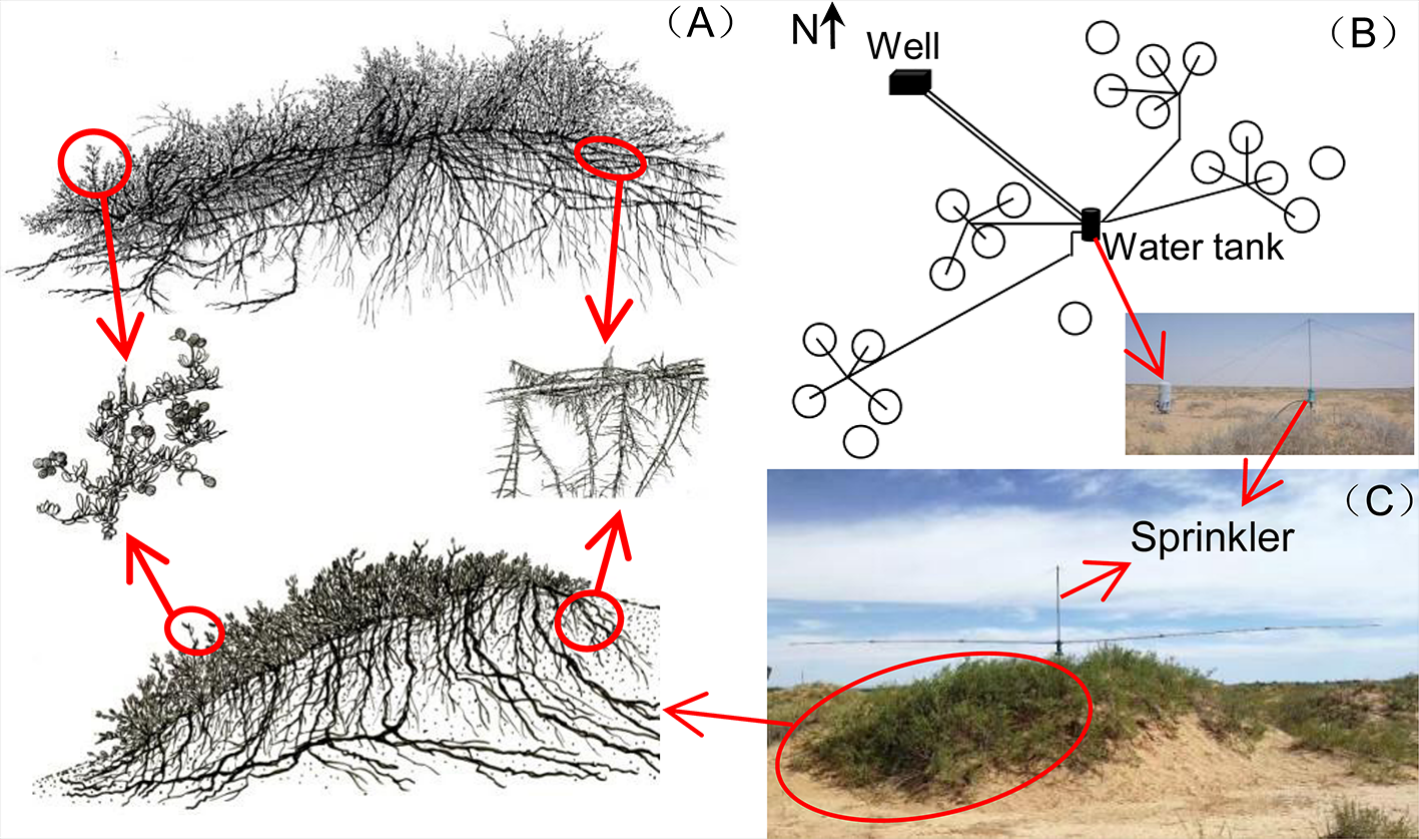
Illustrations of *Nitraria tangutorum* nebkhas above-ground and below-ground structures, cited from Zhang et al., 2015 **(A)**; distribution of the 20 plots and the water addition system **(B)**; sprinkler on nebkhas **(C)**.

This study was conducted at the Desert Ecosystem Water Addition Platform (106°43'E, 40°24'N, 1,050 m above sea level (a.s.l.)) which was set up in Dengkou County, Inner Mongolia, China, in 2008. The long-term mean annual precipitation is approximately 145 mm, 95% of which falls from May to September. The mean annual temperature is 7.6°C, and the mean annual potential evaporation is 2,381 mm. The vegetation is dominated by the shrub species *N. tangutorum*. Other species, such as *Artemisia ordosica*, *Psammochloa villosa*, *Agriophyllum squarrosum*, and *Corispermummon golicum*, can also be found occasionally. *N. tangutorum* has a high tolerance to drought, wind erosion, and sand burial. It is a pioneer species that is widely distributed throughout the northwestern regions of China and plays an important ecological role in the fixing of sand dunes because of its exceptional capabilities to form so-called nebkha dunes, or nebkhas (Zhang et al., 2015). Nebkhas are phytogenic mounds composed of wind-borne sediments within or around shrub canopies (Li et al., 2013). The experiment was performed in a patchy landscape with *N. tangutorum* nebkhas (**Figure 1**) interspersed on hard clay deposited by the Yellow River. The plant cover on these nebkhas was approximately 45–75%. The soil types were sandy soil and gray-brown desert soil (Zhang et al., 2015). The underground water at the experimental site was at a depth below 5 m, which does not affect plant growth. The soil chemical properties at the 0–10 cm depth are shown in **Table 1**.

**Table 1:** Mean ( ± SE) values of soil chemical properties in 0–10 cm deep soil at the end of the 2010 growing season (n = 4).

Treatment	SOC (%)	Soil N (%)	Soil C/N	Soil pH
Ctrl	0.13 ± 0.01(abc)	0.018 ± 0.001(b)	7.80 ± 0.48(a)	8.90 ± 0.11(a)
+25%	0.12 ± 0.01(bc)	0.021 ± 0.002(ab)	5.71 ± 0.71(bc)	9.02 ± 0.12(a)
+50%	0.11 ± 0.01(c)	0.024 ± 0.001(a)	4.57 ± 0.34(c)	8.92 ± 0.11(a)
+75%	0.16 ± 0.01(a)	0.026 ± 0.002(a)	5.94 ± 0.32(bc)	8.66 ± 0.07(a)
+100%	0.15 ± 0.01(ab)	0.024 ± 0.002(a)	6.48 ± 0.62(ab)	8.87 ± 0.06(a)

SOC, soil organic carbon. The same letters in parentheses within each soil property indicate no significant differences between water addition treatments, while different letters denote significant differences (P < 0.05).

### Experimental Design of Water Addition Treatments

A completely random design was used with five water addition treatments: natural rain plus an additional 0% (Control), 25% (+25%), 50% (+50%), 75% (+75%), and 100% (+100%) of local long-term mean annual precipitation (145 mm). Furthermore, four replicates for each treatment were established since 2008 (113 m^2^ per plot, 20 plots in total, **Figure 1B**). The water addition treatments were applied equally every month from May to September, and the additional water amounts were 0, 7.3, 14.5, 21.8, and 29.0 mm each time for the five water addition treatments, respectively. The water was pumped from a well near the plots into a water tank with water meters and then transported to each sprinkler (**Figure 1C**). The sprinklers were installed on the top of each nebkha (plot) *via* an irrigation system (**Figure 1B**). The sprinklers had two automatically rotating spraying arms (6 m in length) that could uniformly sprinkle water over the treatment area. More detailed information on the experimental design and the irrigation system can be found in our previous publication (He et al., 2019). No chemical components were added to the water, and thus, the water used here could be used to simulate natural rainfall (Song et al., 2020).

### Phenology Recording

The phenology observations were conducted from 2012 to 2018 following the standard protocols of Phenological Observation Methodology in China (Wan and Liu, 1979) with minor modifications. Phenology recording was conducted by the same observer from March 2012 to May 2014 and from March 2015 to November 2018, while from May 2014 to November 2014 the recordings were conducted by another observer who had been trained for one month by the first observer. Phenological events for all shrubs in each plot (each nebkha) were recorded every other day.

A series of phenological events, including leaf unfolding (onset, 30%, 50%, and end of leaf unfolding), cessation of new branch elongation (30, 50, 90%), and leaf coloration (80% of leaves turned yellow) were recorded (**Table 2**). Precipitation, air temperature, relative humidity, and evaporation data were recorded by a standard meteorological station near the experimental plots. The soil gravimetric water content (SWC) of the 0–20 cm soil layer was measured using the oven-drying method. SWC measurements were conducted on the day before water-addition treatments and every two days after in May, July, and September in 2012, but only one day each month in 2017 (see details in **Figure 3**).

**Table 2:** Phenological events recorded in this study. All shrubs in each treatment plot (nebkha) were considered and counted.

Leaf unfolding	Onset of leaf unfolding	At least one bud in each nebkha has at least one leaf completely out of the bud, first leaves visible, but not yet at full size.
	30% of leaves unfolded	30% of buds in each nebkha have their leaves out, fully expanded.
	50% of leaves unfolded	50% of buds in each nebkha have their leaves out, fully expanded.
	End of leaf unfolding	More than 90% of buds in each nebkha have their leaves out, fully expanded.
Cessation of new branch elongation	30% of new branches ceased elongating	30% of new branches in each nebkha have their terminal withered and cease elongating
	50% of new branches ceased elongating	50% of the new branches in each nebkha have their terminal withered and cease elongating
	90% of new branches ceased elongating	Over 90% of the new branches in each nebkha have their terminal end withered and ceased elongating
Leaf coloration	80% of leaves turned yellow	Over 80% of the leaves in each nebkha show yellow color.

### Data Processing

The observed dates of phenological events were first transformed to the day-of-year format. The length of the growing season was calculated as the difference between the days when 80% of the leaves had turned yellow and the onset of leaf unfolding. The relative change of the days (Δdays) was used to test the effects of water addition treatments on each event.

(1) $$\Delta \;\text{days}=\frac{1}{n}\sum_{i=0}^{n}\left(\text{da}{\text{y}}_{\mathrm{treat}}-\text{da}{\text{y}}_{\text{Ctrl}}\right)$$

where day_treat_ represents the day for a given event or the length of the growing season under water addition plots, day_Ctrl_ represents the corresponding day in control plots, and n represents the number of experimental years. Then, Δdays <0 indicates that phenological events (growing season length) were advanced (shortened) under water addition, while Δdays >0 indicates that phenological events (growing season length) were delayed (prolonged) under water addition.

This study investigated whether the heat requirement (often expressed as growing degree days, GDD) and chilling day (CD) affected the inter-annual variation of the onset of leaf unfolding in the natural condition. GDD was calculated as the sum of the daily mean temperature exceeding 5°C and below 30°C from Jan 1st to the day before the onset of leaf unfolding (Fu et al., 2013; Anandhi, 2016). CD is the number of days with a daily mean temperature below 0°C from Nov 1st the previous year to the onset of leaf unfolding.

### Statistical Analysis

Simple linear regression analysis was used to determine the inter-annual trends of meteorological factors and phenological events. Pearson correlation was used to analyze the relationships between phenological events and meteorological factors. Linear mixed models were used to examine the effects of water addition treatments, year, and their interactions on phenological events over the seven years (2012–2018). Water and year were used as fixed factors, while plot was used as a random factor. The dependent factor was the timing of different phenological events (Type I Sum of Squares was used). Duncan *post hoc* tests were used to determine pairwise differences for significant effects. Regression analyses were used to determine the relationships between changes in phenological events and water addition amounts or soil water content. Repeated Measurement ANOVA (RMANOVA) analysis was used to test the effects of water addition treatments, time of treatment application, and their interactions on soil water content. One-way ANOVA analyses were used to test the effects of water addition treatments on the timing of different phenological events, separately for each year. Homogeneity of variances was tested by Levene's tests. One-sample Kolmogorov-Smirnov tests were used to validate normality of the data distribution. All these above-mentioned procedures were performed in SPSS (SPSS for Windows, version 20.0, Chicago, IL, USA).

## Results

### Meteorological Factors

Temporal variations in annual mean relative humidity, air temperature, evaporation, and annual precipitation at the study site from 1983 to 2018 are shown in **Figure SI1**. Relative humidity followed a significant increasing trend since 1983 (**Figure SI1A**). No significant temporal trends were found for air temperature (**Figure SI1B**) and evaporation (**Figure SI1C**). Annual precipitation followed increasing trends from 1983 to 1998 and from 1999 to 2018 (**Figure SI1D**).

**Figure 2:**
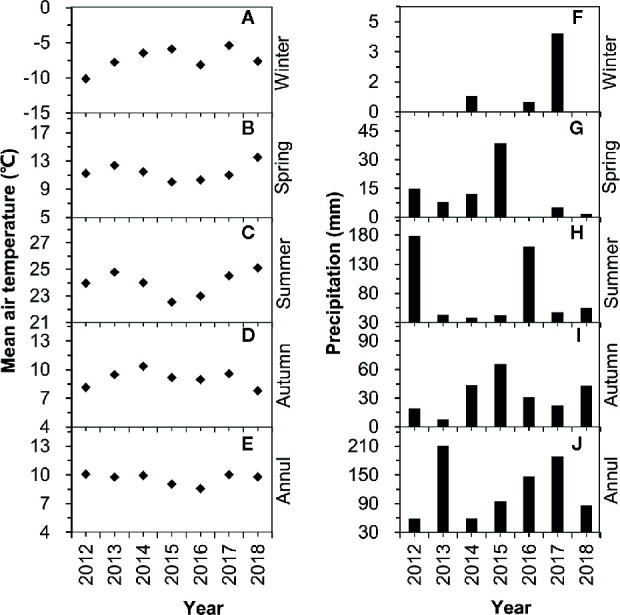
Variations in air temperature **(A**–**E)** and precipitation **(F**–**J)** over the period of 2012–2018.

Seasonal variations in precipitation and air temperature are shown in **Figure 2**. Mean air temperatures in winter, spring, summer, and autumn were −7.34°C, 11.39°C, 23.9 8°C, and 9.09°C, respectively. The mean annual temperature was 9.60°C for 2012–2018, and no significant increasing or decreasing trends were found (**Figures 2A–E**). Winter precipitation only occurred in three of the years from 2012–2018 and the amounts were very low, with less than 1 mm in 2014 and 2016, and less than 5 mm in 2018 (**Figure 2F**). Spring rainfall varied over 2012–2018, with the highest value in 2015 (38.6 mm, including one large rain event with 36.5 mm) and zero in 2016 (**Figure 2G**). Accumulated summer rainfall exceeded 35 mm in all seven years (2012–2018), with the highest value in 2012 (178.5 mm) and the lowest value in 2014 (38.5 mm) (**Figure 2H**). There was a large variation in autumn rainfall (2012–2018), with the highest value in 2015 (65.6 mm) and the lowest value in 2013 (7.6 mm) (**Figure 2I**). Annual precipitation in 2012, 2013, 2014, 2015, 2016, 2017, and 2018 was 213.3 mm, 59.1 mm, 95.2 mm, 147 mm, 189.2 mm, 86.0 mm, and 59.1 mm, respectively (**Figure 2J**). Based on the deviation from the long-term mean, 2012 and 2016 were “above-average” (i.e., wet) years, 2013 was an “ultra below-average” (i.e., extremely dry) year, while 2014, 2017, and 2018 were “below-average” (i.e., dry) years, and 2015 was an “average” year.

### Variation Trends of Phenological Events for 2012-2018

The dates of phenological events (in days of the year) under different water addition treatments are shown in **Table SI1**. No significant advancing or delaying trends were found for almost all phenological events under all treatments for 2012–2018 (all *P >*0.05) with only one exception (the onset of leaf unfolding) (**Table SI2**). The onset of leaf unfolding dates showed significantly earlier trends for 2012–2018 for all treatments (all *P <*0.05, **Figure SI2**). Among all meteorological factors, only relative humidity (RH) was significantly negatively correlated with the dates of onset of leaf unfolding under all treatments (all *P <*0.05, **Table SI3**). Almost no significant relationships were found between all other phenological events and meteorological factors (**Table SI3**). No significant effects of temperature (winter, spring, summer, autumn, and annual) were detected for any of the phenological events in this study (all *P >*0.05, **Table SI3**).

### Changes in Soil Water Content

**Figure 3:**
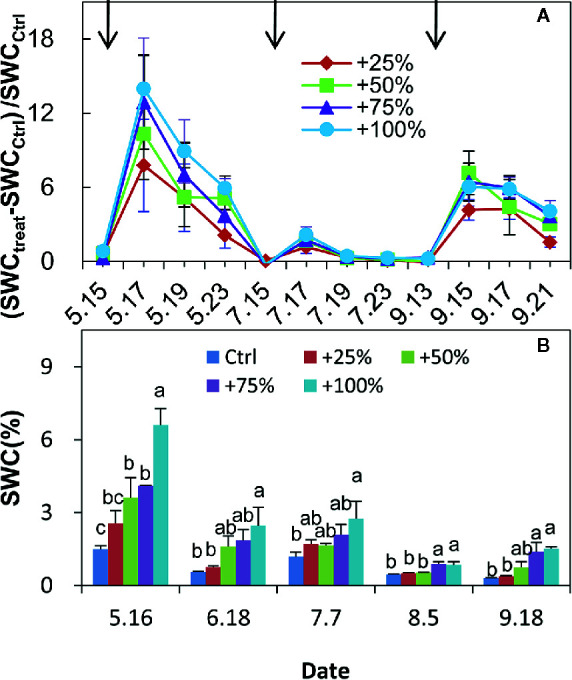
Relative changes of 0–20 cm soil water content (SWC) compared with control (Ctrl) in +25, +50, +75, and +100% water addition treatment plots in spring (May), summer (July), and autumn (September) in 2012 **(A)**. Variation in 0–20 cm SWC during the growing season (May to September) of 2017 **(B)**. SWC_Ctrl_ represents SWC in control plots. SWC_treat_ represents the SWC in water addition treatment plots. Arrows in **(A)** represent the water addition treatment application time.

RMANOVA analysis showed that both the amount of added water and time had significant effects on soil water content (SWC) (*P <*0.01), and their interaction was also significant (*P <*0.01). The SWC significantly changed after water addition treatments each month (**Figures 3A, B**). The change in magnitude depended on the amount and time of water added. Larger water addition resulted in a larger response magnitude (**Figures 3A, B**). The relative changes of soil water contents after water addition treatments were highest in spring, followed by autumn, and were lowest in summer (**Figures 3A, B**).

### Changes in Spring Phenology

Linear Mixed Model analysis showed that water addition treatments had significant effects on the occurrence of 30% of leaves unfolded and the end of leaf unfolding (all *P <*0.05, **Table 3**), and had marginally significant effects on the occurrence of 50% of leaves unfolded (*P* = 0.08, **Table 3**). Year affected all events significantly (all *P <*0.05, **Table 2**). There was no interaction between water addition treatment and year (all *P >*0.05, **Table 3**).

**Table 3:** Results (*P*-values) of Linear Mixed Model (MIXMOD) analysis on the fixed effects of water addition treatments (water), year, and their interactions on the phenological events and the length of the growing season from 2012–2018.

Phenological events		Water	Year	Water × Year
Leaf unfolding	Onset of leaf unfolding	0.16	<0.01	0.20
	30% of leaves unfolded	0.03	<0.01	0.65
	50% of leaves unfolded	0.08	<0.01	0.73
	End of leaf unfolding	<0.01	<0.01	0.32
Cessation of new branch elongation	30% of new branches ceased elongating	0.07	<0.01	0.24
	50% of new branches ceased elongating	0.85	<0.01	0.80
	90% of new branches ceased elongating	0.01	<0.01	0.53
Leaf coloration	80% of leaves turned yellow	0.02	<0.01	0.41
The length of the growing season		0.01	<0.01	0.53

**Figure 4:**
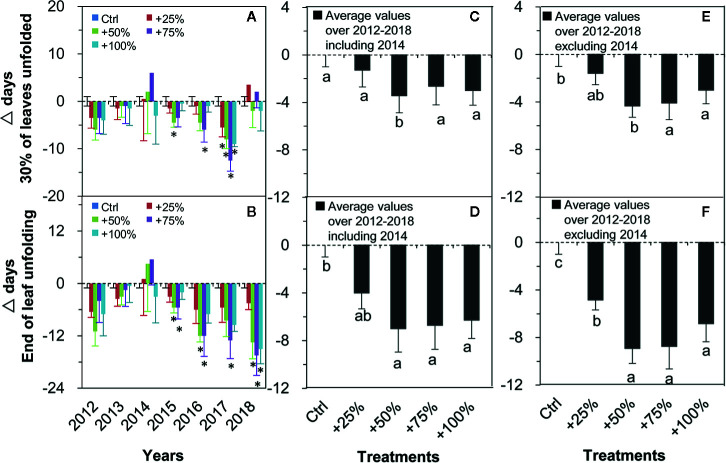
Relative changes (△days; mean ± SE) of the two spring events of *N. tangutorum* after water addition treatments (+25% to +100%) compared with the control (Ctrl). Positive and negative values represent delayed and advanced days, respectively. **A, C, E** represent the 30% of leaves unfolded event. **B, D, F** represent the End of leaf unfolding event. * indicates significant differences at the *P <*0.05 level compared with values in control plots.

Two of the four spring events (leaf unfolding), 30% of leaves unfolded (**Figure 4A**) and the end of leaf unfolding (**Figure 4B**), showed consistent directional shifts (both advanced) in all four water addition treatment plots in all seven years except for 2014 (**Figure 4**). On average over the seven years (2012–2018), the occurrence of 30% of leaves unfolded events in +25, +50, +75, and +100% treatments were advanced by 1.29, 3.43, 2.64, and 3.00 d, respectively, which was significantly different from control in the +50% treatment (*P <*0.05, **Figure 4C**). On average in six years of the study (2012–2018, excluding 2014), the occurrence of 30% of leaves unfolded events in +25, +50, +75, and +100% treatments were advanced by 1.58, 4.33, 4.08, and 3.00 d, respectively. Significant differences from the control occurred in +75 and +100% treatments (all *P <*0.05, **Figure 4E**). On average over all seven years (2012–2018), the occurrences of the end of leaf unfolding event in +25, +50, +75, and +100% treatments were advanced by 4.00, 7.00, 6.71, and 6.29 d, respectively, and significant differences from the control occurred in +50, +75, and +100% treatments (all *P <*0.05, **Figure 4D**). On average in six years of the study (2012–2018, excluding 2014), the occurrences of the end of leaf unfolding event in +25, +50, +75, and +100% treatments were advanced by 4.83, 8.92, 8.75, and 6.83 d, respectively. Significant differences from the control occurred in +25, +50, +75, and +100% treatments (all *P <*0.05, **Figure 4F**).

### Changes in Autumn Phenology

Linear Mixed Model analysis showed that water addition treatments had significant effects on the cessation of new branch elongation (90%) and 80% of leaves turned yellow (all *P <*0.05, **Table 3**), while the effect on the cessation of new branch elongation (30%) was marginally significant (*P* = 0.07, **Table 3**). Year affected all events significantly (all *P <*0.05, **Table 3**). No interaction was found between water addition treatments and year (all *P >*0.05, **Table 2**).

**Figure 5:**
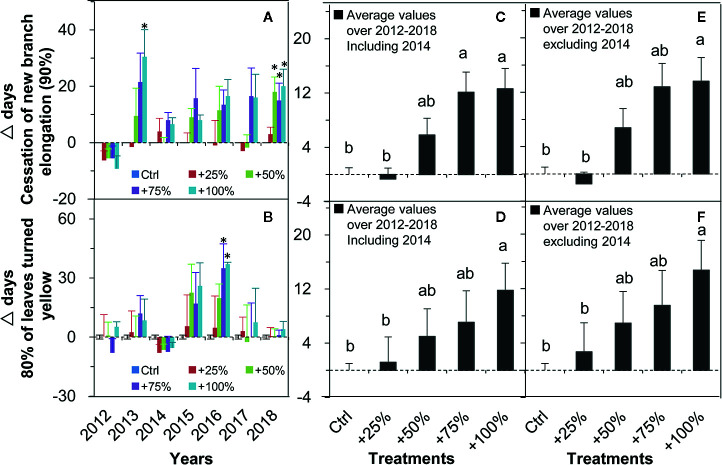
Relative changes (△days; mean ± SE) of the two autumn events of *N. tangutorum* after water addition treatments (+25% to +100%) compared with the control (Ctrl). Positive and negative values represent delayed and advanced days, respectively. **A, C**, and **E** represent the cessation of new branch elongation. **B, D,** and **F** represent 80% of leaves turned yellow. * indicates significant differences at the *P <*0.05 level compared with values in control plots.

The cessation of new branch elongation (90%) was delayed in all four water addition treatments and all seven years (2012–2018) with one exception in 2012 (**Figure 5A**). On average over the seven years (2012–2018), the cessation of new branch elongation (90%) in +50, +75, and +100% treatments were delayed by 5.82, 12.11, and 12.61 d, respectively. Significant differences from control occurred in +75 and +100% treatments (all *P <*0.05, **Figure 5C**). On average in six years of the study (2012–2018, excluding 2014), the cessation of new branch elongation (90%) in +50, +75, and +100% treatments were delayed by 6.79, 12.79, and 13.63 d, respectively. Significant differences from the control occurred in +100% treatment (*P <*0.05, **Figure 5E**).

The dates when 80% of leaves turned yellow were delayed in all four water addition treatments and in all seven years in almost all cases except for 2012 and 2014 (**Figure 5B**). On average over the seven years (2012–2018), the occurrence of 80% of leaves turning yellow was delayed in +25, +50, +75, and 100% treatments by 1.18, 5.00, 7.07, and 11.82 d, respectively. A significant difference from the control occurred in the +100% treatment (**Figure 5D**). On average over six years of the study (2012–2018, excluding 2014), the occurrence of 80% of leaves turned yellow was delayed in +25, +50, +75, and 100% treatments by 2.71, 6.92, 9.50, and 14.71 d, respectively. A significant difference from the control occurred in the +100% treatment (*P <*0.05, **Figure 5F**).

### Changes in the Length of the Growing Season

Linear Mixed Model analysis showed that both water addition treatments and year had significant effects on the length of the growing season (*P <*0.05, **Table 3**), and there was no interaction between them.

**Figure 6:**
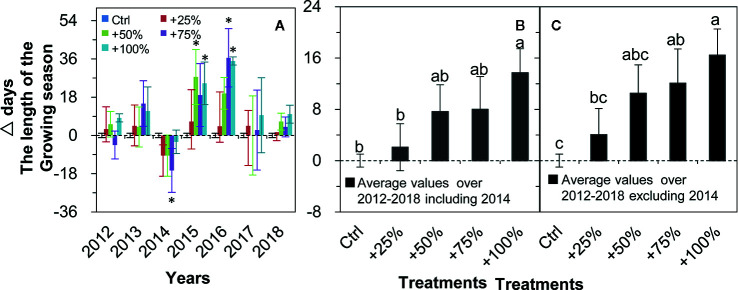
Relative changes (△days; mean ± SE) in the growing season length of *N. tangutorum* after water addition treatments (+25% to +100%) compared with the control (Ctrl). Positive and negative values represent prolonged and shortened days, respectively. **A–C** represent the length of the growing season. * indicates significant differences at the *P <*0.05 level compared with values in control plots.

The growing season was prolonged in all four water addition treatments and in all seven years (2012–2018) in all cases except for 2012 and 2014 (**Figure 6A**). On average over the seven years (2012–2018), the growing season was prolonged in +25, +50, +75, and 100% treatments by 2.11, 7.64, 8.00, and 13.68 d, respectively, and a significant difference from control occurred in the +100% treatment (*P <*0.05, **Figure 6B**). On average in six years of the study (2012–2018, excluding 2014), the growing season was prolonged in +25, +50, +75, and 100% treatments by 4.04, 10.50, 12.08, and 16.46 d, respectively, and significant differences from the control occurred in +75 and +100% treatments (all *P <*0.05, **Figure 6C**).

### Relationships Between Phenological Events and Water Addition Amounts

Spring phenology. Significant decreases were observed in quadratic relationships between the timing of the onset of leaf unfolding and soil water content in 2012 and 2017 (all *P <*0.05, **Figure SI3A**), and between the timing of 30% of leaves unfolding (*P <*0.05, **Figure SI3B**), 50% of leaves unfolding (*P <*0.05, **Figure SI3C**), and end of leaf unfolding (*P <*0.05, **Figure SI3D**) and soil water content in 2017.

**Figure 7:**
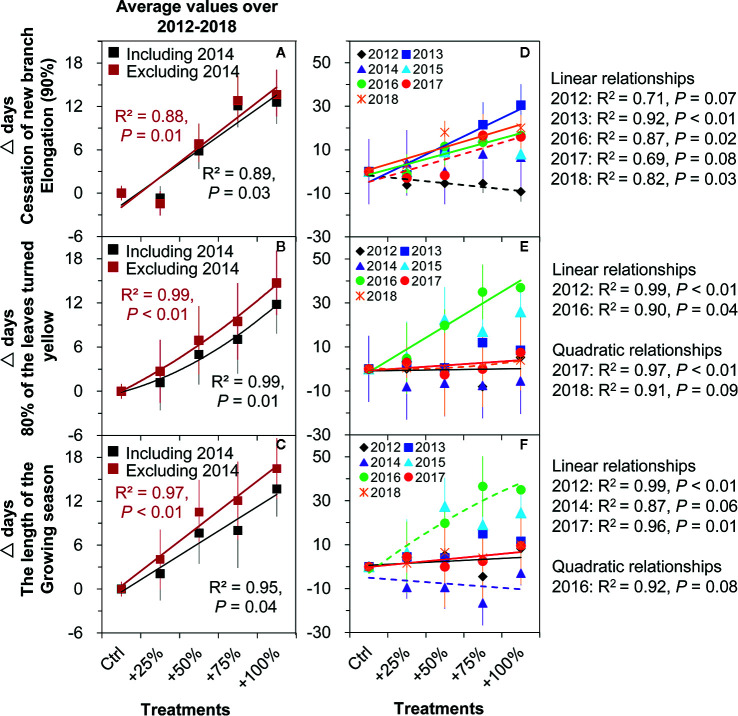
Correlations between the amount of water addition (Ctrl), +25, +50, and +100%) and the changes (△days) of the phenological events of *N. tangutorum*. The solid lines indicate *P* ≤0.05, and the dashed lines indicate 0.05< *P <*0.1. **A, D** represent cessation of new branch elongation. **B, E** represent 80% of the leaves turned yellow. **C, F** represent the length of the growing season.

Autumn phenology. Significant positive linear relationships were found between the timing of cessation of new branch elongation (90%) and amount of water addition on average from 2012–2018 (all *P <*0.05, **Figure 7A**). Among the seven years, significant or marginally significant relationships were determined in five years including 2012 (*P* = 0.07), 2013 (*P <*0.01), 2016 (*P* = 0.02), 2017 (*P* = 0.08), and 2018 (*P* = 0.03) (**Figure 7D**). However, a significantly increased quadratic relationship between 80% of leaves turned yellow and water addition amount was determined on average over the period of 2012–2018 without an apparent threshold (all *P <*0.05, **Figure 7B**). Among the seven years, significant relationships were found in four years, such as linear relationships in 2012 (*P <*0.01) and 2016 (*P* = 0.04), and increasing quadratic relationships in 2017 (*P <*0.01) and 2018 (*P* = 0.09) (**Figure 7E**).

Growing season length. Significant positive linear relationships were found between the growing season length and amount of water addition on average from 2012–2018 (all *P <*0.05, **Figure 7C**). Among the seven years, significant relationships were found in four years, such as linear relationships in 2012 (*P <*0.01), 2014 (*P* = 0.06), and 2017 (*P* = 0.01), and an increasing quadratic relationship in 2016 (*P* = 0.08) (**Figure 7F**).

## Discussion

### Impact of Water Addition Treatment on Spring Phenology

Han et al. (2015) conducted a two-year water addition experiment in a temperate desert steppe of northwestern China, and found that water addition treatment did not affect the timing of the green-up of dominant species but delayed the senescence time of selected species by 1.93–9.57 days in one year (Han et al., 2015). In the present study, the water addition treatment advanced spring phenology (30% of leaves unfolded, end of leaf unfolding) and delayed the autumn phenology (80% of leaves turned yellow) in the desert shrub *N. tangutorum*. This is partly consistent with the results reported by Han et al., 2015. In general, in the present study, increasing the water addition amount only affected the variation magnitudes of phenological events but not their shifting direction.

Gebauer and Ehleringer (2000) irrigated five dominant cold desert shrub species in southern Utah during different seasons. Based on stable isotope data, they found that all species derived less than 10% of the irrigation water in spring (May) and used most of the water in autumn (September). This result implies that shrubs mainly use soil water in early spring (Golluscio et al., 1998). Compared to control plots, the additionally added water could help to recharge spring soil in water addition treatment plots, and if more water was added, the deeper soil could also be recharged (Ogle and Reynolds, 2004). As a result, in the present study, it is reasonable to attribute the advanced spring phenology to the increased antecedent soil moisture condition caused by the long-term water addition treatment even though the data are not available.

### Impact of Water Addition Treatment on Autumn Phenology

Autumn phenology regulates multiple aspects of ecosystem function (e.g., altering carbon/nitrogen cycling and biotic interactions), along with associated feedback to the climate system (Keenan and Richardson, 2015; Xie et al., 2018). Delayed autumn phenology lengthened the duration of the growing season of *N. tangutorum*. According to the growing season length calculation formula (time of 80% of leaves turned yellow minus time of onset of leaf unfolding), it is reasonable to conclude that autumn events contributed more to the lengthening of the growing season than the early spring event in this study. The lengthened growing season possibly implies a longer carbon uptake period and increased accumulation of photosynthates (Zhang et al., 2012). This might outweigh the potential risk of frost damage because of cold spells caused by the earlier leaf unfolding of *N. tangutorum* in spring. The delayed cessation of branch elongation in the present study further confirmed this conclusion. The prolonged branch elongation stage may improve carbohydrate allocation to root production, which, in turn, would enhance water and nutrient utilization in *N. tangutorum*.

The start of autumn phenology is a highly regulated process that involves the sequential degradation of macromolecules and the extensive salvage of nutrients (Fracheboud et al., 2009). Environmental factors, such as temperature and photoperiod (Fracheboud et al., 2009), precipitation (Zhou and Jia, 2016), frost and moisture condition (rainfall patterns) (Xie et al., 2015), heat and drought stress (Xie et al., 2018), as well as spring phenology (Fu et al., 2014b; Keenan and Richardson, 2015), were found to significantly affect inter-annual variation in autumn phenology. No significant correlations were found between spring and autumn phenology in the present study. The delayed autumn phenology found in this study may be partly because the water addition treatment enhanced the activities of photosynthetic-related enzymes of *N. tangutorum* (Bao et al., 2017; He et al., 2019) and slowed the speed of chlorophyll degradation during leaf senescence (Fracheboud et al., 2009).

### Effects of Year on Plant Phenology

The shifting directions under water addition treatments were consistent for almost all events except for several exceptions, such as 30% of leaves unfolded in 2014 and 2018, the end of leaf unfolding in 2014, and autumn phenology in both 2012 and 2014. GDD, CD, precipitation, and insolation have complex interactions in their effects on spring vegetation green-up phenology (Fu et al., 2012; Fu et al., 2014a; Fu et al., 2015). In 2012, 2013, 2014, 2015, 2016, 2017, and 2018 in control plots, GDD was 429.9 heat unit (HU), 392.4 HU, 303.85 HU, 269.00 HU, 195.40 HU, 241.30 HU, and 279.45 HU, respectively. In 2012, 2012, 2013, 2014, 2015, 2016, 2017, and 2018 in control plots, CD was 112, 111, 112, 107, 108, 99, and 106 d, respectively. GDD and CD in 2012 and 2014 were neither too high nor too low from 2012 cessation 2018, suggesting that heat and chilling requirements were sufficiently met to break dormancy in the two years and should not be related to the exceptions. In addition to temperature, the antecedent soil moisture plays an important role in regulating the effects of water addition on plant phenology as it may either diminish or amplify the effects of water increase on plant growth and photosynthesis (Reynolds et al., 2004). The soil water condition in the early springs of 2014 and 2018 might be dry because the soil water could not have been well recharged by the low autumn rainfalls of the previous years (Mazer et al., 2015; Moore et al., 2015; Cleverly et al., 2016) of only 7.6 mm in 2013 and 22.2 mm in 2017. Moreover, the low spring rainfall in both years (2014, 12.1 mm; 2018, 1.7 mm) further aggravated the situation. Lower water addition treatments (+25, +50, and +75%) were insufficient for the alleviation of natural drought. As a result, the delay of the spring events in +25, +50, and +75% water addition treatment plots in 2014 and 2018 can mainly be attributed to variations in the antecedent soil water availability (Ogle and Reynolds, 2004; Reynolds et al., 2004) and the complicated interactions with environmental factors (Xie et al., 2015). The advanced autumn phenology in 2012 might be related to the water stress caused by several large rainfall events during that year (Xie et al., 2015). However, the advanced autumn phenology in 2014 might be related to an extremely dry summer which resulted in heat and drought stress on plant phenology even in the water addition treatment plots (Xie et al., 2015; Xie et al., 2018). Alternatively, it might be affected by the change of the phenology observer (**Figures 4**–**7**, these observations have not affected the conclusion of this manuscript; therefore, the data from 2014 were included). It is reasonable to speculate that climate change projections of an earlier and longer growing season in response to the increasing precipitation in desert ecosystems remains elusive for years when plants face consecutively extreme drought and water-logging stress (Adams et al., 2015).

### Responses of Phenological Events to Water Addition Treatments

Many studies have shown that water plays an important role in driving plant phenology, such as budburst (Hayden et al., 2010), green-up (Zhang et al., 2005; Liu et al., 2013), flowering (Crimmins et al., 2010; Lesica and Kittelson, 2010; Crimmins et al., 2011; Crimmins et al., 2013; Kigel et al., 2013; Sakkir et al., 2014; Huang et al., 2018), and fruiting (Lotfi and Mohamed, 2006; Galindo et al., 2014), in water-limited ecosystems. However, most of these studies are focused on natural conditions, while the relationship between water increase and phenological events based on water addition experiments has not been explored, nor has the effects of water addition on branch phenology of desert plants. The magnitude of spring phenology advance under water addition treatments might be limited by the acquisition of nutrients by roots, while root growth will be limited by the availability of photosynthates from leaves (Linares et al., 2012). In addition, leaf lifespan (autumn phenology) is associated with nutrient remobilization (especially nitrogen) and storage of photosynthates (Kozlowski and Pallardy, 1997). There are trade-offs between leaf unfolding and carbohydrate allocation to roots, as well as between leaf coloration and nutrient remobilization. That may be why non-linear patterns were found between phenology and water availability. The present study captured the nonlinear nature of plant responses to increased water availability (Ogle and Reynolds, 2004), suggesting that predictions of phenological events in the future should not be based only on linear trends.

## Conclusion

The findings of the present study suggest that the phenological pattern of the desert shrub species *N. tugutorum* was significantly influenced by increased precipitation and soil water availability. This offers insight on the effects of other environmental factors (in addition to temperature) on phenology. However, the soil water content during non-growing seasons (late autumn, winter, and early spring) was not monitored. Given the important role of the early spring water availability, which affects early spring phenological events, the soil water content should be monitored in all seasons in the future. Since different species have different phenological responses to climate change (Xie et al., 2018), more desert species should be studied in future experiments.

## Data Availability Statement

All datasets generated for this study are included in the article/**Supplementary Material**.

## Author Contributions****

FB contributed to manuscript writing, data analysis, and data collecting. ML, YC, ZX, JL, and BY contributed to the data collecting. QL and BW contributed to manuscript writing and data collecting.

## Funding

This research was supported by Funds of the National Key Research and Development Project (Grant Nos. 2016YFC0500806 and 2016YFC0501004), the National Natural Science Foundation of China (Grant No. 31400421), and the Surplus Funds of Institute of Desertification Studies, CAF (Grant Nos. IDS2008JY-3 and IDS2018JY-9).

## Conflict of Interest

The authors declare that the research was conducted in the absence of any commercial or financial relationships that could be construed as a potential conflict of interest.
