# Supplementary material for: Water Addition Prolonged the Length of the Growing Season of the Desert Shrub Nitraria tangutorum in a Temperate Desert
Source: Front Plant Sci. 2020 Jul 21;11:1099. doi: 10.3389/fpls.2020.01099 (PMC7386313; doi:10.3389/fpls.2020.01099)
Supplement: Supplementary file 1 [file DataSheet_1.pdf]

Table SI1 Starting day of each phenological event for *Nitraria tangutorum* over the period of 2012-2018 for each water addition treatment, Ctrl (control), +25%, +50%, +75%, and +100%.

Phenological events	Treatment	2012	2013	2014	2015	2016	2017	2018	Average values over 2012-2018
Onset of leaf unfolding	Ctrl	117.50±2.06 a	114.50±0.50a	102.50±3.86ab	108.50±1.50ab	101.00±0.00a	105.50±0.50a	99.50±0.50a	107.00±1.36a
	+25%	114.5±2.22a	112.50±1.50a	104.00±4.76ab	107.50±1.50ab	101.50±0.50a	104.00±0.00a	98.50±1.26a	106.07±1.27a
	+50%	113.00±2.16a	111.00±3.16a	105.50±3.30ab	103.50±1.50b	101.00±0.82a	103.00±1.29a	93.50±0.96a	104.36±1.35a
	+75%	114.00±2.65a	111.50±2.99a	111.50±2.63a	106.50±2.63ab	99.50±2.36a	103.00±2.08a	96.50±3.95a	106.07±1.51a
	+100%	114.50±3.10a	111.50±2.22a	100.00±0.82b	110.00±1.83a	103.00±1.15a	103.50±1.50a	93.50±0.96a	105.14±1.44a
30% of leaves unfolded	Ctrl	122.00±3.00a	117.50±0.96a	113.00±6.24a	114.50±0.50a	111.00±0.00a	124.00±2.31a	105.50±4.57a	115.36±1.58a
	+25%	118.50±2.87a	116.00±1.91a	113.50±7.09a	113.00±0.58a	110.00±0.58a	118.50±1.50ab	109.00±1.73a	114.07±1.24a
	+50%	116.00±2.38a	116.50±2.36a	115.00±3.00a	110.00±0.82a	106.50±1.71ab	116.00±2.00b	103.50±1.26a	111.93±1.16b
	+75%	118.50±3.40a	116.50±3.86a	119.00±1.29a	111.00±1.73a	105.00±2.58b	111.50±3.77b	107.50±3.40a	112.71±1.40a
	+100%	118.00±3.32a	116.00±3.79a	110.00±0.82a	114.00±1.15a	110.00±1.29a	115.00±1.73b	103.50±2.36a	112.36±1.17a
50% of leaves unfolded	Ctrl	128.00±4.73a	120.00±1.29a	108.50±5.12a	117.00±0.58a	115.00±0.00a	130.00±3.46a	115.00±1.30a	119.07±1.69a
	+25%	123.50±5.19a	118.00±1.91a	110.00±6.16a	115.50±0.50a	114.50±0.50a	123.00±1.29ab	113.00±1.73a	116.79±1.41a
	+50%	120.00±2.52a	118.50±2.36a	112.00±2.94a	113.50±0.50a	111.00±1.83a	121.00±2.38ab	108.50±0.96a	114.93±1.11a
	+75%	125.50±5.74a	119.50±4.79a	115.50±1.50a	114.50±1.26a	109.50±2.99a	116.50±4.92b	108.00±6.16a	115.57±1.79a
	+100%	121.50±3.95a	119.00±4.76a	106.50±0.96a	116.00±1.15a	115.00±1.15a	121.00±2.38ab	107.50±2.06a	115.21±1.42a
End of leaf unfolding	Ctrl	138.00±4.50a	130.50±1.70a	124.50±6.29a	125.00±1.29a	131.00±1.63a	140.00±2.44a	137.00±2.38a	132.28±1.57a
	+25%	131.50±5.19a	127.00±1.82a	125.50±6.55a	122.00±1.15a	125.00±1.83ab	134.50±1.7ab	132.50±1.25a	128.28±1.4ab
	+50%	127.00±1.63a	127.50±2.21a	129.00±4.65a	119.50±1.26a	119.00±2.16b	131.50±3.2ab	123.50±1.7a	125.29±1.22b
	+75%	134.00±6.02a	129.00±4.54a	130.00±2.16a	119.50±2.75a	119.00±4.55b	127.00±4.93b	120.50±5.79a	125.57±1.85b
	+100%	131.00±4.69a	130.00±4.72a	121.50±0.95a	123.00±1.73a	124.00±3.32ab	130.50±2.87ab	122.00±5.35a	126.00±1.45b

30% of new branches ceased elongating	Ctrl	236.00±2.08a	187.50±0.49a	220.50±2.98a	171.00±1.15a	205.00±1.91a	188.00±4.54b	172.00±1.15c	197.14±4.44a
	+25%	235.00±1.15a	190.00±1.29a	219.50±4.19a	171.50±0.49a	205.50±1.70a	181.50±0.95b	172.50±1.50c	196.50±4.38a
	+50%	236.00±1.00a	190.50±2.06a	217.75±4.36a	176.50±0.95a	208.00±1.63a	186.00±3.65ab	174.00±2.00bc	198.39±4.18a
	+75%	231.50±2.21a	188.50±2.62a	218.00±0.81a	176.50±4.34a	209.25±1.49a	192.75±10.84ab	183.50±5.91ab	200.00±3.96a
	+100%	231.00±1.82a	187.50±0.95a	221.00±1.29a	175.00±0.81a	212.75±1.03a	185.00±2.51a	188.50±3.94a	200.12±3.84a
50% of new branches ceased elongating	Ctrl	241.50±2.36a	195.50±2.62a	236.00±2.82a	176.50±0.95b	219.00±2.82a	203.00±3.00a	177.50±2.06b	207.00±4.74a
	+25%	239.50±1.50a	195.00±1.41a	233.50±2.87a	177.50±0.49b	217.00±0.81a	200.50±0.95a	178.00±1.82b	205.85±4.48a
	+50%	241.50±0.49a	199.50±4.50a	232.00±3.82a	186.50±1.50a	222.50±2.98a	203.50±3.77a	184.00±6.00ab	209.93±4.18a
	+75%	237.00±2.58a	196.00±4.12a	233.50±1.70a	183.50±5.12ab	228.50±7.63a	215.00±10.87a	202.00±13.63a	213.64±4.46a
	+100%	235.00±1.82a	193.50±0.95a	238.50±2.62a	186.00±2.38a	226.50±2.06a	210.00±5.11a	200.00±3.91ab	212.79±3.84a
90% of new branches ceased elongating	Ctrl	252.50±3.20a	213.00±1.15b	247.50±1.50a	189.50±0.95	239.50±4.99b	223.00±2.94a	251.00±5.8b	230.86±4.34b
	+25%	246.25±1.60ab	211.50±0.49b	251.50±3.94a	189.50±3.30	238.50±4.86b	220.00±1.29a	254.00±4.08bc	230.18±4.4b
	+50%	247.00±0.81ab	222.50±8.99ab	247.50±2.21a	198.50±3.77	251.00±4.69ab	221.25±2.39a	269.00±1.73a	236.68±4.46ab
	+75%	247.00±3.91ab	234.50±10.14ab	255.50±2.06a	205.25±10.98	253.00±6.00ab	239.50±12.68a	266.00±6.87ab	242.96±4.46a
	+100%	243.25±1.65b	243.5±9.94a	254.00±2.94a	197.50±1.50	256.00±1.00a	239.00±10.55a	271.00±1.29a	243.46±4.50a
80% of leaves turned yellow	Ctrl	294.50±6.71a	278.00±10.54a	294.50±2.50a	258.00±11.86a	258.50±2.75b	274.00±9.88a	292.00±4.83a	278.50±3.87b
	+25%	294.50±4.97a	280.50±7.54a	286.50±2.22a	263.50±7.97a	263.25±14.21b	277.00±8.29a	292.50±1.89a	279.68±3.45b
	+50%	295.25±2.46a	278.50±5.50a	288.00±2.16a	280.50±3.50a	278.25±8.26ab	271.50±9.00a	292.50±1.71a	283.50±2.37ab
	+75%	286.50±7.71a	290.00±12.12a	287.00±5.45a	275.00±6.35a	293.50±12.69a	274.00±12.12a	293.00±4.51a	285.57±3.41ab
	+100%	299.75±4.27a	286.50±8.66a	289.00±3.42a	284.00±1.83a	295.50±3.50a	281.50±9.74a	296.00±1.41a	290.32±2.22a
The length of the growing season	Ctrl	177.00±6.28a	163.50±10.66a	192.00±6.06a	149.50±11.18b	157.50±2.75b	168.50±9.57a	192.50±4.99a	171.50±3.96b
	+25%	180.00±4.26a	168.00±8.29a	182.50±3.30ab	156.00±6.98ab	161.75±14.27ab	173.00±8.26a	194.00±2.45a	173.61±3.48b
	+50%	182.25±4.52a	167.50±8.10a	182.50±3.95ab	177.00±2.65a	177.25±8.69ab	168.50±9.18a	199.00±2.08a	179.14±2.81ab
	+75%	172.50±9.84a	178.50±14.93a	175.50±4.79b	168.50±8.18ab	194.00±14.28a	171.00±14.12a	196.50±8.42a	179.50±4.25ab
	+100%	185.25±6.24a	175.00±10.54a	189.00±3.32ab	174.00±2.16a	192.50±4.57a	178.00±10.85a	202.50±1.89a	185.18±2.88a

The same letters within each phenological event indicate no significant differences between water addition treatments, while different letters denote significant differences ($P < 0.05$).

Table SI2 R^2 and *P* values of simple linear regressions of phenological events against year sequence of 2012-2018.

	Ctrl		+25%		+50%		+75%		+100%	
	R^2	*P*	R^2	*P*	R^2	*P*	R^2	*P*	R^2	*P*
Onset of leaves unfolding	0.68	0.02	0.81	0.01	0.88	0.00	0.89	0.00	0.64	0.03
30% of leaves unfolding	0.22	0.29	0.30	0.20	0.47	0.09	0.65	0.03	0.51	0.07
50% of leaves unfolding	0.09	0.51	0.23	0.27	0.36	0.16	0.71	0.02	0.38	0.14
End of leaves unfolding	0.08	0.55	0.09	0.52	0.04	0.66	0.52	0.07	0.18	0.35
30% of new branches ceased elongating	0.42	0.11	0.48	0.08	0.47	0.09	0.31	0.20	0.26	0.24
50% of new branches ceased elongating	0.33	0.18	0.34	0.17	0.36	0.16	0.07	0.56	0.10	0.50
90% of new branches ceased elongating	0.00	0.96	0.01	0.85	0.05	0.64	0.06	0.58	0.07	0.58
80% of leaves turned yellow	0.06	0.59	0.05	0.64	0.08	0.53	0.00	0.90	0.03	0.72
Growing season length	0.01	0.83	0.03	0.69	0.12	0.45	0.27	0.23	0.21	0.31

Table SI3 Coefficients of Pearson Correlations between meteorological factors and phenological events over 2012-2018

**Meteorological factors**		**Onset of leaf unfolding**					**30% of leaves unfolded**					**50% of leaves unfolded**					**End of leaf unfolding**				
		**Ctrl**	**+25%**	**+50%**	**+75%**	**+100%**	**Ctrl**	**+25%**	**+50%**	**+75%**	**+100%**	**Ctrl**	**+25%**	**+50%**	**+75%**	**+100%**	**Ctrl**	**+25%**	**+50%**	**+75%**	**+100%**
Accumulated precipitation	Winter	-0.21	-0.24	-0.11	-0.19	-0.19	0.54	0.45	0.35	-0.08	0.17	0.63	0.52	0.39	-0.01	0.39	0.44	0.52	0.59	0.12	0.35
	Spring	0.37	0.40	0.27	0.40	0.50	0.16	0.16	0.16	0.26	0.38	-0.02	0.03	0.10	0.26	0.19	-0.52	-0.55	-0.28	-0.06	-0.14
	Summer	0.26	0.24	0.27	0.06	0.33	0.14	0.06	-0.09	-0.11	0.23	0.14	0.20	-0.14	0.07	0.25	0.33	0.09	-0.29	0.14	0.28
	Autumn	-0.47	-0.44	-0.52	-0.29	-0.33	-0.51	-0.54	-0.52	-0.29	-0.45	-0.51	-0.52	-0.36	-0.31	-0.54	-0.54	-0.49	-0.54	-0.59	-.805*
	Annual	0.10	0.07	0.09	-0.16	0.15	0.32	0.24	0.23	-0.23	0.28	0.15	0.08	0.01	-0.26	0.34	0.21	0.12	0.19	-0.11	0.50
Mean Air temperature	Winter	-0.45	-0.43	-0.38	-0.27	-0.34	0.01	-0.05	0.03	-0.18	-0.15	-0.03	-0.11	0.11	-0.23	-0.09	-0.30	-0.15	0.15	-0.36	-0.30
	Spring	-0.12	-0.18	-0.27	-0.17	-0.46	-0.38	-0.20	-0.18	0.09	-0.48	-0.15	-0.21	-0.16	-0.11	-0.49	0.33	0.50	0.32	0.16	-0.04
	Summer	0.01	-0.07	-0.09	-0.06	-0.34	0.02	0.17	0.15	0.21	-0.21	0.26	0.17	0.14	0.06	-0.14	0.56	0.75	0.66	0.39	0.30
	Autumn	-0.08	0.05	0.28	0.38	0.06	0.27	0.25	0.55	0.39	0.25	0.12	0.22	0.66	0.40	0.33	-0.57	-0.40	0.39	0.21	-0.06
	Annual	0.34	0.30	0.31	0.43	0.01	0.43	0.61	0.58	0.69	0.20	0.66	0.64	0.62	0.60	0.26	0.44	0.68	.88**	.78*	0.43
Relative humidity		**-.83***	**-.90****	**-.94****	**-.94****	**-.80***	-0.46	-0.55	-0.69	-.81*	-0.72	-0.30	-0.48	-0.60	-.84*	-0.61	0.28	0.29	-0.20	-0.72	-0.42
**Meteorological factors**		**30% of new branches ceased elongating**					**50% of new branches ceased elongating**					**90% of new branches ceased elongating**					**80% of leaves turned yellow**				
		**Ctrl**	**+25%**	**+50%**	**+75%**	**+100%**	**Ctrl**	**+25%**	**+50%**	**+75%**	**+100%**	**Ctrl**	**+25%**	**+50%**	**+75%**	**+100%**	**Ctrl**	**+25%**	**+50%**	**+75%**	**+100%**
Accumulated precipitation	Winter	-0.06	-0.17	-0.14	-0.05	-0.20	0.06	0.03	-0.01	0.16	0.09	-0.07	-0.09	-0.22	0.01	-0.01	-0.11	-0.12	-0.62	-0.58	-0.56
	Spring	-0.16	-0.15	-0.12	-0.24	-0.27	-0.23	-0.21	-0.19	-0.45	-0.36	-0.66	-0.65	-0.71	-.84*	-.90**	-0.29	-0.31	0.04	-0.62	-0.35
	Summer	0.64	0.65	0.69	0.69	0.69	0.58	0.59	0.65	0.63	0.57	0.48	0.38	0.39	0.27	0.21	-0.02	0.04	0.32	0.38	.77*
	Autumn	-0.37	-0.36	-0.36	-0.35	-0.27	-0.37	-0.36	-0.35	-0.34	-0.22	-0.27	-0.18	-0.16	-0.30	-0.41	-0.25	-0.32	0.15	-0.29	-0.14
	Annual	-0.40	-0.41	-0.41	-0.43	-0.51	-0.32	-0.34	-0.35	-0.35	-0.44	-0.45	-0.48	-0.39	-0.22	-0.05	-0.47	-0.41	-.83*	-0.11	-0.53
Mean Air temperature	Winter	-0.59	-0.64	-0.64	-0.61	-0.66	-0.47	-0.49	-0.53	-0.46	-0.42	-0.56	-0.48	-0.53	-0.39	-0.38	-0.37	-0.45	-0.65	-0.65	-.88**
	Spring	-0.20	-0.18	-0.24	-0.17	-0.09	-0.24	-0.24	-0.29	-0.13	-0.16	0.44	0.48	0.57	0.59	0.67	0.68	0.73	0.44	0.53	0.26
	Summer	-0.09	-0.10	-0.15	-0.07	-0.07	-0.09	-0.10	-0.16	0.02	-0.06	0.42	0.42	0.43	0.56	0.64	0.69	0.74	0.19	0.29	0.06
	Autumn	0.11	0.08	0.07	0.03	-0.03	0.23	0.22	0.18	0.07	0.14	-0.33	-0.28	-0.45	-0.26	-0.25	-0.16	-0.33	-0.54	-0.35	-0.69
	Annual	0.26	0.22	0.19	0.24	0.16	0.24	0.23	0.17	0.20	0.18	0.34	0.32	0.15	0.27	0.25	.83*	.85*	0.34	-0.14	-0.05
Relative humidity		-0.65	-0.69	-0.69	-0.56	-0.51	-0.57	-0.59	-0.60	-0.27	-0.31	0.03	0.09	0.22	0.25	0.26	-0.25	-0.22	-0.29	-0.06	-0.17

*represents *P* value is significant at the 0.05 level, ** represents *P* value is significant at the 0.01 level.

Winter, Spring, Summer, and Autumn in this table represent Dec1-Feb29, Mar1-May31, Jun1-Aug31, and Sep1-Nov31, respectively.
