# Supplementary material for: Water Addition Prolonged the Length of the Growing Season of the Desert Shrub Nitraria tangutorum in a Temperate Desert
Source: Front Plant Sci. 2020 Jul 21;11:1099. doi: 10.3389/fpls.2020.01099 (PMC7386313; doi:10.3389/fpls.2020.01099)
Supplement: Supplementary file 2 [file DataSheet_2.pdf]

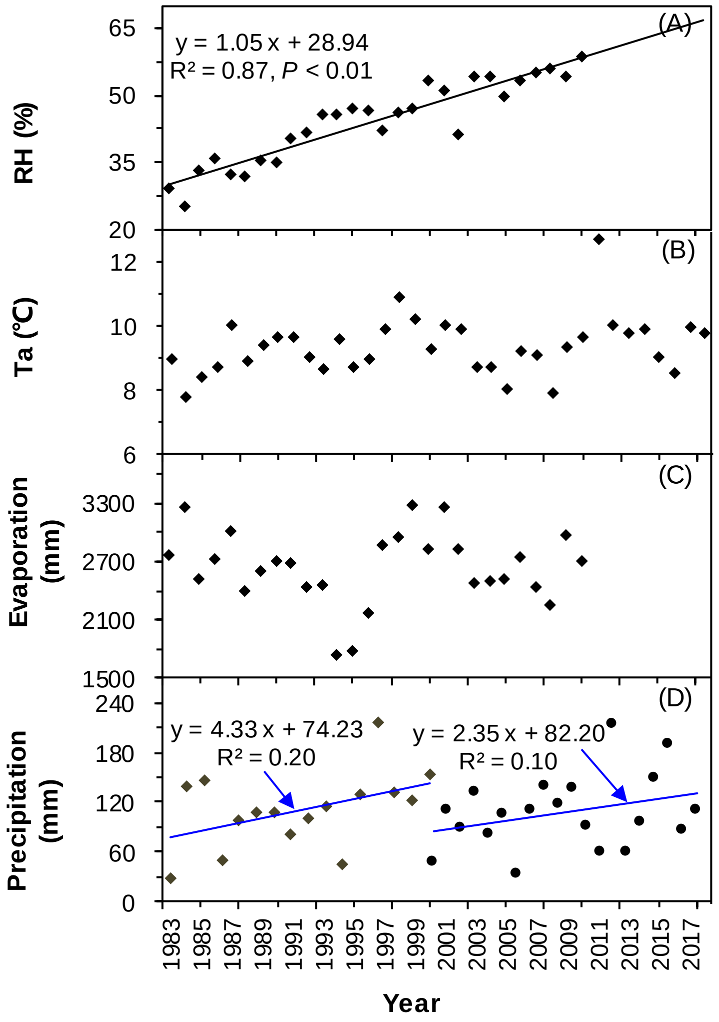

Figure SI1 Temporal changes in annual mean relative humidity (RH) (A), air temperature (B), evaporation (C), and annual precipitation (D) from 1983–2018 at the study site. Solid black squares and circles in graph D represent annual mean precipitation in 1983–1998 and 1999–2018, respectively.

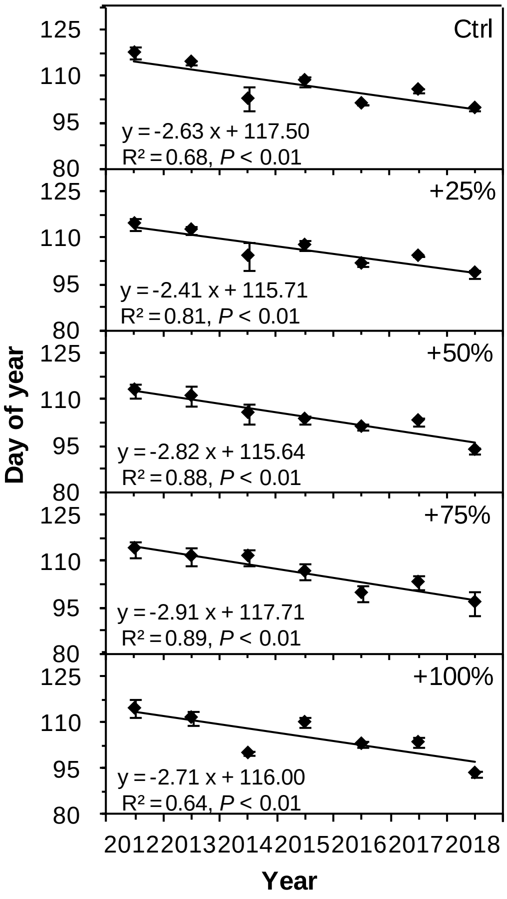

Figure SI2 Temporal changes in the onset of leaf unfolding in *Nitraria tantutorum* under water addition treatments, calculated using simple linear regression over the period of 2012-2018 for the study site.

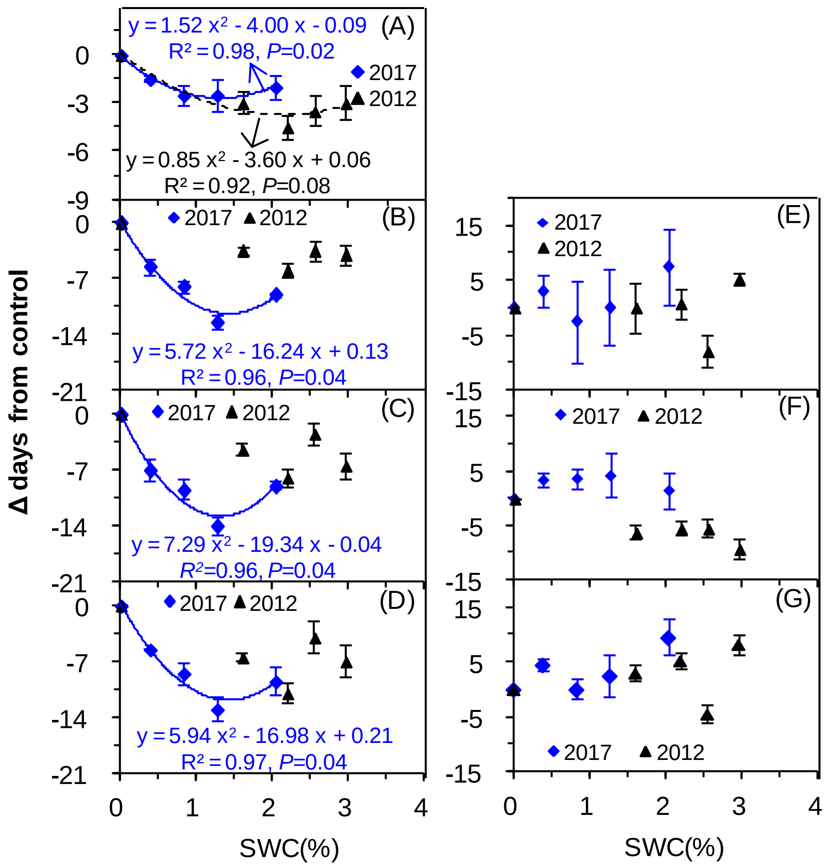

Figure SI3 The correlation between phenological events of *N. tangutorum* and soil water content. A, onset of leaf unfolding; B, 30% of leaves unfolding; C, 50% of leaves unfolding; D, end of leaf unfolding; E, 80% of leaves turned yellow; F, the cessation of new branch elongation (90%); G, the length of the growing season.
